# Adenosine-induced Asystole during AVM Embolization: A Case Series

**DOI:** 10.1007/s00062-021-01035-z

**Published:** 2021-06-14

**Authors:** V. Hellstern, P. Bhogal, M. Aguilar Pérez, M. Alfter, A. Kemmling, E. Henkes, O. Ganslandt, H. Henkes

**Affiliations:** 1 Neuroradiological Clinic, Klinikum Stuttgart, Stuttgart, Germany; 2 The Royal London Hospital, Barts Health NHS Trust, Whitechapel Road, E1 1BB London, UK; 3 Neurosurgical Clinic, Klinikum Stuttgart, Stuttgart, Germany; 4 Medical Faculty, University Duisburg-Essen, Essen, Germany

**Keywords:** Adenosine, Asystole, AVM, Embolization, Resection

## Abstract

**Background:**

Adenosine induced cardiac standstill has been used intraoperatively for both aneurysm and arteriovenous malformation (AVM) surgery and embolization. We sought to report the results of adenosine induced cardiac standstill as an adjunct to endovascular embolization of brain AVMs.

**Material and Methods:**

We retrospectively identified patients in our prospectively maintained database to identify all patients since January 2007 in whom adenosine was used to induce cardiac standstill during the embolization of a brain AVM. We recorded demographic data, clinical presentation, Spetzler Martin grade, rupture status, therapeutic intervention and number of embolization sessions, angiographic and clinical results, clinical and radiological outcomes and follow-up information.

**Results:**

We identified 47 patients (22 female, 47%) with average age 42 ± 17 years (range 6–77 years) who had undergone AVM embolization procedures using adjunctive circulatory standstill with adenosine. In total there were 4 Spetzler Martin grade 1 (9%), 9 grade 2 (18%), 15 grade 3 (32%), 8 grade 4 (18%), and 11 grade 5 (23%) lesions. Of the AVMs six were ruptured or had previously ruptured. The average number of embolization procedures per patient was 5.7 ± 7.6 (range 1–37) with an average of 2.6 ± 2.2 (range 1–14) embolization procedures using adenosine. Overall morbidity was 17% (*n* = 8/47) and mortality 2.1% (*n* = 1/47), with permanent morbidity seen in 10.6% (*n* = 5/47) postembolization. Angiographic follow-up was available for 32 patients with no residual shunt seen in 26 (81%) and residual shunts seen in 6 patients (19%). The angiographic follow-up is still pending in 14 patients. At last follow-up 93.5% of patients were mRS ≤2 (*n* = 43/46).

**Conclusion:**

Adenosine induced cardiac standstill represents a viable treatment strategy in high flow AVMs or AV shunts that carries a low risk of mortality and permanent neurological deficits.

## Introduction

Adenosine, an endogenous purine nucleoside, suppresses sinus and atrioventricular (AV) node conduction and hence can induce transient AV blockade. At low doses (3–12 mg) it is a commonly used intravenous antiarrhythmic agent; however, at higher doses it can trigger ventricular asystole and resultant hypotension or circulatory standstill.

The initial report of adenosine induced hypotension and asystole in neurosurgery were by Pile-Spellman et al. [1] for the endovascular embolization of a high-flow arteriovenous malformation (AVM). Subsequently, numerous reports have documented the use of this technique in neurosurgery, principally for the treatment of aneurysms [2–10] but also for AVMs [1, 11–15].

Here we report our results of adenosine induced ventricular asystole from a series of 44 patients.

## Methods

### Patient Population

We performed a retrospective analysis of our prospectively maintained databases to identify all patients who underwent endovascular embolization, either partial or complete, for an AVM. The data set included patients with unruptured and ruptured AVMs as well as those that were found incidentally or presented with nonhemorrhagic neurological symptoms. We identified all patients who were treated between January 2007 and January 2020.

For each patient we recorded demographic data, clinical presentation, Spetzler Martin grade, rupture status, therapeutic intervention and number of embolization sessions, angiographic and clinical results, clinical and radiological outcomes and follow-up information.

### Endovascular Treatment

Informed consent was obtained before the procedure from all patients or their legal representatives unless in the case of emergency treatment. Patients were explicitly informed that induced asystole is an established but off-label use of adenosine. It was explained that amongst the other procedural risks, severe and even life-threatening arrhythmia and the need for emergency electrical cardioversion may result. Patients were also informed that within the personal experience of the senior author with several hundred adenosine asystoles, no clinically relevant complications had occurred.

The preparation of the patients included a routine 12-lead electrocardiograph (ECG) examination and in the case of any known or newly detected cardiac abnormality a complete cardiac work-up. At the beginning of the endovascular procedure defibrillator pads were placed on the patient and a defibrillator was connected ready for use. Via the common femoral vein a 4 F venous access a diagnostic catheter (Tempo4 vertebral, Cordis, Santa Clara, CA, USA) was introduced under fluoroscopy with the tip of the catheter positioned at the level of the right atrium. Invasive intra-arterial blood pressure measurement was used by default.

All treatments were performed with the patient under general anesthesia via the right common femoral artery using a short 6 F sheath and a 6 F guide catheter. In patients without or outside the acute phase of intracranial hemorrhage, anticoagulation was achieved with a 5000 IU bolus dose of unfractionated heparin at the start of the procedure and subsequent 1000 IU bolus doses every hour to maintain the activated clotting time between 2–2.5 times the baseline. Standard biplane DSA runs were acquired and served to select target vessels for the embolization procedure. In general:

- a reduction of the volume of the AVM nidus,
- an occlusion of as many feeding arteries as possible,
- a reduction of the arteriovenous shunt volume,
- no compromise of the venous drainage of the AVM,

were the goals of the endovascular procedures.

Standard flow directed microcatheters (Marathon/Mirage, Medtronic; Dublin, Ireland, Magic 1.2, Balt Extrusion, Montmorency, France) were used to catheterize the target vessels. The injection of 0.3–0.5 ml Imeron 300 (Bracco Imaging, Konstanz, Germany) with a 2 ml syringe enabled the angioarchitecture of the arteriovenous connection to be visualized. In the case of many small-caliber vessels (plexiform nidus) or a few medium-caliber vessels (microfistulous nidus), embolization was carried out without induced asystole. In the case of a large-caliber high flow arteriovenous connection (macrofistulous nidus), embolization under induced asystole was considered.

In all cases n‑butyl-cyanoacrylate (nBCA, Glubran2, GEM; histoacryl, B. Braun, Melsungen, Germany) was used as the occlusive liquid agent. Iodized oil (Lipiodol ultra fluid, Guerbet GmbH, Sulzbach, Germany) was added in equal parts in order to make the occlusive agent radiopaque and to slightly delay the polymerization for better control. Histoacryl polymerizes faster than Glubran2 and was used if this appeared to be advantageous. Glubran2 has a CE mark for intra-arterial embolization, which is not the case for histoacryl. Histoacryl, however, is identical with Trufill nBCA (Cordis), which is approved by the FDA for the embolization of brain AVMs. The mixture of either Glubran2 or histoacryl with Lipiodol is an off-label use. The nBCA and Lipiodol were drawn into a 1 ml syringe and manually shaken for at least 2 min.

Asystole was induced by a bolus injection of 36 mg adenosine (Adrekar, Sanofi-Aventis) through a 4 F venous catheter at the level of the right atrium. This dose is used as standard and is independent from age and body weight. In the vast majority of patients 10–20 s of asystole is achieved. We do not start an infusion of sodium nitroprusside nor do we give hypotensive agents during the procedure.

Before the injection of adenosine the microcatheter is thoroughly flushed with a 5% glucose solution. While the anesthesiologist injects the adenosine bolus the interventionist fills the dead space of the microcatheter with nBCA/Lipiodol. Once the electric cardiac standstill begins the interventionist counts to 8, and then slowly starts to inject the embolic liquid. Visual control is achieved by high frame rate (e.g., 6/s) DSA or, for radioprotection purposes, under high frame rate (e.g., 30/s) fluoroscopy. The speed and volume of glue injection has to be calibrated in a way that avoids both venous passage and proximal reflux. Fixation of the microcatheter by the glue cast is avoided by rapid removal of the microcatheter. A few millimeters glue reflux around the tip of the microcatheter can be accepted since the microcatheter can still be separated from the polymer without difficulty.

## Results

**Fig. 1:**
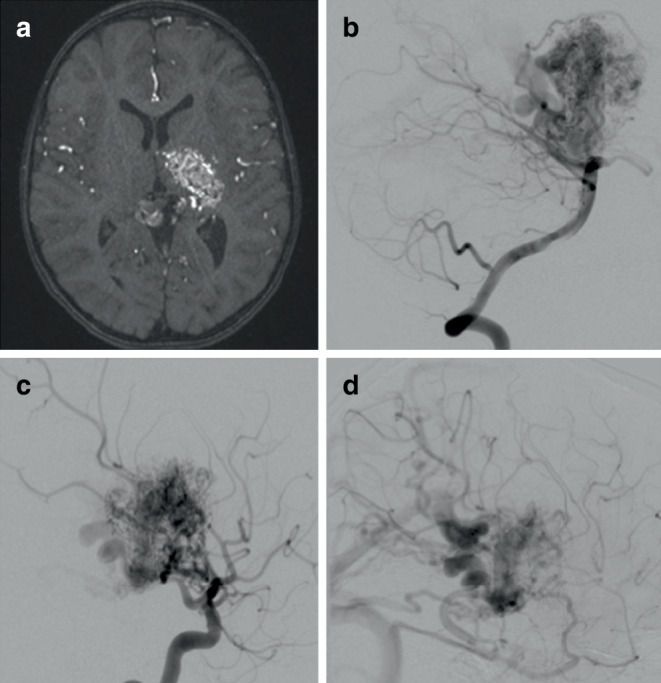
A patient with a left thalamic and ganglion AVM as demonstrated on the axial TOF MRA (**a**). Supply to the AVM was derived from the posterior circulation (**b**) and the anterior circulation (**c**) after contrast injection into the vertebral and internal carotid arteries respectively. On the delayed angiographic images, predominantly deep venous drainage was seen (**d**)

**Fig. 2:**
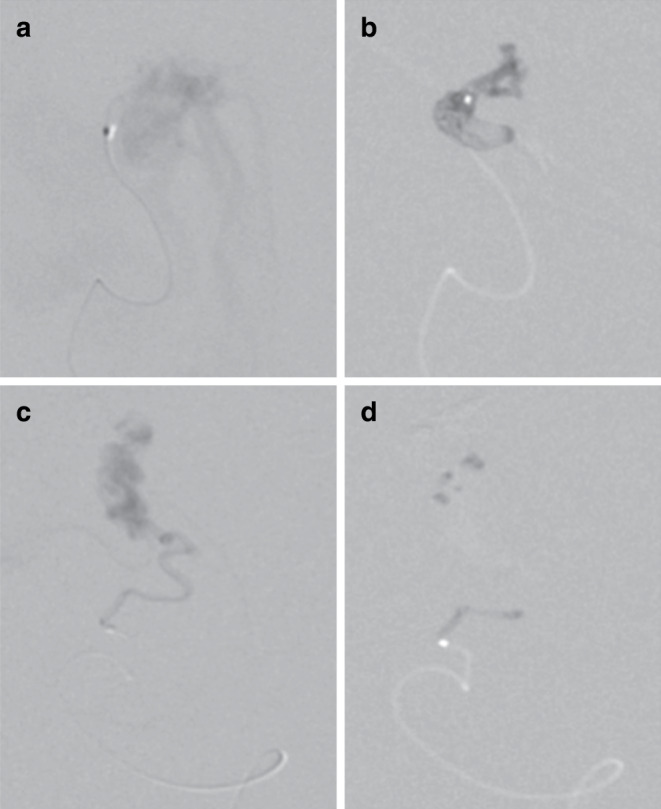
Multiple embolizations were undertaken over a period of several months. Microcatheter angiography via the anterior choroidal artery (**a**) demonstrated rapid shunting. Embolization under asystole (16 s) resulted in good nidal penetration and glue cast formation (**b**). A further embolization via the posterolateral choroidal branch (**c,** **d**) but without induced asystole resulted in poor nidal penetration

**Fig. 3:**
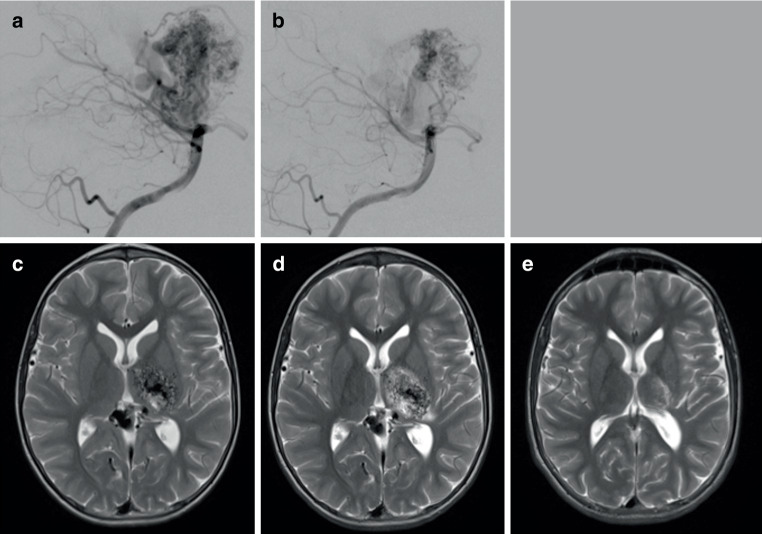
The angiographic appearances pre-embolization (**a**) and post-multiple embolization sessions including those performed under asystole demonstrated a significant reduction in the volume of the nidus (**b**). An axial T2-weighted magnetic resonance imaging (MRI) post-embolization demonstrated continued shunting but no major infarction (**c**). Gamma knife radiosurgery was performed to complete the treatment, which caused some perinidal edema. (**d**) At year 4 post-radiosurgery there was complete obliteration of the AVM (**e**)

We identified 47 patients (22 female, 47%) with average age of 42 ± 17 years (range 6–77 range) who had undergone AVM embolization procedures using adjunctive circulatory standstill with adenosine (Table 1). In total there were 4 Spetzler Martin grade 1 (9%), 9 grade 2 (18%), 15 grade 3 (32%), 8 grade 4 (18%), and 11 grade 5 (23%) lesions. Of the AVMs six were ruptured or had previously ruptured, whereas 41 were unruptured (Figs. 1, 2 and 3).

**Table 1:** Patient details, AVM grading, operative details, complications, radiographic and clinical outcome

Demographics			AVM status		Embolization Procedure				Surgical Procedure		Functional status preoperatively and postoperatively		Radiation procedure		Radiographic and clinical outcome	
Patient No.	Age	Gender	Rupture Status	Spetzler Martin grade	Total number of Embolizations	Embolic Material used	Total number of Adenosine injections	Symptomatic Complications Post-Embolisation	Surgical Resection	Symptomatic Complications post-surgery	mRS pre-operative	mRS postEmbol	Radiation Treatment	Symptomatic Complications post-radiotherapy	Outcome at last angiogram	mRS last follow up
1	47	f	Unruptured	II	3	Histoacryl/Glubran	3	–	Y	–	0	0	N	–	No shunt	0
2	52	f	Unruptured	IV	3	Histoacryl/Glubran	5	–	Y	–	0	0	N	–	No shunt	1
3	51	f	Unruptured	II	2	Histoacryl/Glubran	5	–	Y	–	0	0	N	–	No shunt	0
4	70	f	Ruptured	I	2	Glubran	1	–	N	–	1	0	N	–	No shunt	0
5	26	m	Unruptured	IV	4	Glubran	2	–	Y	Motor aphasia	0	0	N	–	No shunt	2
6	6	f	Unruptured	III	7	Glubran2/Phil/Onyx18	3	–	N	–	0	0	N	–	No shunt	0
7	27	f	Ruptured	III	2	Glubran	1	–	N	–	3	3	Y	–	Pending	0
8	40	m	Unruptured	IV	5	Glubran/Onyx	3	–	N	–	0	0	Y	–	Pending	0
9	37	f	Unruptured	III	4	Glubran	3	–	Y	–	0	0	N	–	No shunt	0
10	31	f	Unruptured	III	1	Glubran	3	–	Y	–	0	0	N	–	No shunt	0
11	45	m	Unruptured	I	2	Glubran	1	–	N	–	0	0	Y	–	No shunt	0
12	32	m	Unruptured	I	2	Glubran	1	–	Interruption of AV shunt but no resection	–	0	0	N	–	No shunt	0
13	33	m	Unruptured	III	1	Glubran	2	–	Interruption of AV shunt but no resection	–	0	0	N	–	No shunt	0
14	24	m	Unruptured	IV	7	Glubran	3	Improving Hemiparesis	N	–	0	1	N	–	Residual shunt	1
15	31	m	Ruptured	V	17	Glubran	2	–	N	–	0	0	N	–	Pending	0
16	43	m	Unruptured	II	1	Glubran	3	–	Interruption of AV shunt but no resection	–	0	0	N	–	No shunt	0
17	55	f	Unruptured	V	2	Glubran/Histoacryl	7	–	N	–	?	?	N	–	Pending	?
18	70	m	Unruptured	IV	5	Glubran	14	Death	N	–	0	6	N	–	NA	6
19	48	m	Ruptured	III	3	Glubran	1	–	N	–	0	0	Y	–	Pending	0
20	27	f	Unruptured	II	1	Glubran	1	–	Y	–	0	0	N	–	No shunt	0
21	69	f	Unruptured	III	2	Glubran	1	–	N	–	0	0	N	–	Pending	0
22	36	f	Ruptured	IV	1	Glubran	1	–	Y	Hemianopia	0	0	N	–	Pending	1
23	58	f	Unruptured	III	3	Glubran	3	Fine motor control and walking difficutly	N	–	0	2	N	–	No shunt	1
24	10	m	Ruptured	II	2	Glubran	2	–	Y	–	4	1	N	–	No shunt	1
25	51	f	Unruptured	III	5	Glubran/Onyx	1	Transient hemiparesis	N	–	0	0	Y	–	Pending	0
26	14	m	Unruptured	V	4	Glubran	1	–	N	–	2	2	Y	–	Pending	2
27	77	f	Unruptured	II	1	Glubran	2	–	Y	–	1	1	N	–	No shunt	1
28	43	m	Unruptured	III	1	Glubran	1	–	N	–	0	0	N	–	Pending	0
29	42	m	Unruptured	I	1	Glubran	2	–	Y	–	0	0	N	–	No shunt	0
30	63	m	Unruptured	IV	9	Glubran	1	–	N	–	0	0	N	–	Pending	0
31	42	f	Ruptured	II	1	Glubran	1	–	N	–	0	0	Y	–	No shunt	0
32	24	m	Unruptured	V	10	Glubran	1	–	N	–	0	0	N	–	Pending	0
33	18	m	Unruptured	III	6	Glubran	2	Transient hemiparesis	N	–	1	1	Y	Edema–Transient hemiasthesia	Residual shunt	0
34	51	m	Unruptured	V	25	Glubran	1	Improving Hemiparesis	N	–	0	2	Y	–	Residual shunt	2
35	74	m	Ruptured	II	2	Glubran	1	Transient aphasia	N	–	4	2	N	–	Residual shunt	1
36	32	f	Unruptured	V	7	Glubran	3	Improving Hemiparesis	N	–	0	0	Y	–	Pending	0
37	58	f	Unruptured	III	7	Glubran	1	–	Y	–	0	0	N	–	No shunt	0
38	57	m	Ruptured	III	2	Histoacryl	6	–	N	–	3	2	N	–	No shunt	2
39	54	m	Unruptured	III	3	Glubran2/Histoacryl	3	–	Y	–	0	0	N	–	Residual shunt	0
40	37	f	Unruptured	V	3	Glubran2/Histoacryl	4	–	Y	Hemiplegia	2	2	N	–	No shunt	5
41	17	f	Unruptured	IV	6	Glubran 2	3	–	N	–	0	0	N	–	Pending	0
42	39	f	Unruptured	V	37	Glubran/Histoacryl	3	–	Y	–	0	1	Y	–	Residual shunt	1
43	55	f	Ruptured	V	20	Glubran/Histoacryl	4	–	Interruption of AV shunt but no resection	Meningitis, seizures	0	0	N	–	No shunt	0
44	24	m	Unruptured	V	28	Glubran/Onyx	1	–	Y	Hemianopia	0	1	N	–	No shunt	1
45	30	m	Unruptured	V	3	Glubran	4	–	Interruption of AV shunt but no resection	–	1	1	N	–	No shunt	1
46	46	m	Unruptured	II	1	Glubran	3	–	Interruption of AV shunt but no resection	–	1	1	N	–	No shunt	1
47	41	m	Unruptured	III	4	Glubran/Histoacryl	1	Bleeding during last embolisation (normal perfusion pressure breakthrough)	Interruption of AV shunt but no resection	–	1	5	N	–	No shunt	4

*mRS* modified Rankin Scale, *AV* Arteriovenous

The average number of embolization procedures per patient was 5.7 ± 7.6 (range 1–37) with an average of 2.6 ± 2.2 (range 1–14) embolization procedures performed with the use of adenosine. In the vast majority of cases Glubran or Glubran 2 was used but in 13 cases 2 different embolic agents were used.

Overall morbidity was 17% (*n* = 8/47) and mortality 2.1% (*n* = 1/47), with permanent morbidity seen in 10.6% (*n* = 5/47) post-embolization. Of these complications three were transient hemiparesis and in a further three cases there was an improving hemiparesis. In one case hemorrhage post-embolization occurred. In total 23 patients underwent surgery post-embolization with interruption of the residual shunt but no resection of the AVM in 7 cases and in 16 cases there was removal of the AVM. There were 5 complications postoperatively (5/23, 21.7%).

In total 11 patients underwent radiosurgery after embolization and in 1 patient embolization, surgery, and radiosurgery were performed. Of the 11 patients who underwent radiosurgery there was a single complication (edema and transient hemianesthesia 9.1%).

Angiographic follow-up was available for 32 patients with no residual shunt seen in 26 (81%) and residual shunts seen in 6 patients (19%). The angiographic follow-up is still pending in 14 patients.

At baseline, 43 patients were recorded as mRS ≤2 (93.5%, *n* = 46) with clinical follow-up available in 46 patients of whom 43 patients were recorded as mRS ≤2 (93.5%).

## Discussion

This article presents the largest series to date on the use of adenosine induced cardiac standstill in the embolization of high flow AVMs. The results suggest that the embolization of AVMs under cardiac standstill with adenosine carries an overall risk of morbimortality close to 20%; however, the risk of death is low and the majority of complications do not result in permanent disability. Virtually all patients in our cohort were mRS ≤2 at last follow-up.

Pile-Spellman et al. [1] were the first to report the use of adenosine induced cardiac stand still in 1999. In their case, a 40-year-old woman presented with a small intracranial hemorrhage and new onset tonic-clonic seizures and was found to have a 4 × 5 cm AVM in the medial anterior occipital lobe on MRI. Angiography revealed a very high-flow AVM with arterial supply principally via the posterior cerebral artery with some collaterals from the middle cerebral artery as well as transdural supply from the middle meningeal vessels. At the initial embolization the transdural supply was occluded. At the second session they felt that systemic hypotension to a mean arterial pressure (MAP) of 40–50 mm Hg would not be sufficient to prevent venous passage of the embolic agent given the extremely high-flow nature of the AVM. Therefore, it was decided to induce a cardiac pause of approximately 10–15 s during which the NBCA could be safely injected. Once in position with a Magic 1.8 F microcatheter (Balt Extrusion, Montmorency, France) the authors initially tried an IV bolus dose of 12 mg of adenosine via the femoral sheath; however, this resulted in atrioventricular blockade (AVB) of only 2 cardiac cycles. Subsequently, dosages of 30 mg and 48 mg were tried; however, neither resulted in a cardiac pause of sufficient length and a dose of 64 mg was needed to induce asystole for between 10–15 s. During the test doses contrast medium was injected through the microcatheter to ensure there was no flow reversal in the feeding artery of the AVM during the asystolic period. The authors reported a drop in the systemic MAP from 80 mm Hg to 20 mm Hg and during the cardiac pause nBCA (1:1 mixture of nBCA with Lipiodol) was injected resulting in obliteration of the AVM. There were no neurological or cardiovascular complications and the patient was discharged 2 days postprocedure with a plan for further embolization prior to definitive surgical resection. The authors also noted that there was a rebound in the systemic MAP after the asystolic period and that this could theoretically cause migration of glue into the draining vein and hence rupture of the AVM depending on the angioarchitecture. For this reason, they recommended mild systemic vasodilation and with nitroprusside coupled with a gradual return to baseline pressures and in this case the authors premedicated the patient with 30 mg of nimodipine. The authors noted that one advantage of this technique over the use of systemic agents, such as esmolol, to induce hypotension is the prolonged effect on the cardiovascular system that these agents can have. In contrast adenosine has a half-life of approximately 10 s and in the event of a failure to revert to sinus rhythm in a timely fashion external ventricular pacing can be instigated until the adenosine induced AV blockade terminates. A rebound increase of the arterial blood pressure after the adenosine induced asystole may occur. The uncontrolled distal migration of the polymerized glue is unlikely since nBCA is different from ethylene-vinyl alcohol copolymer (Onyx, Medtronic) in becoming firmly attached to the vessel wall during polymerization. Distal migration of incompletely polymerized glue and hemorrhagic complications induced by undue arterial hypertension remain a concern. It is therefore recommended to avoid any increase in the systolic arterial blood pressure over 120 mm Hg. The intravenous injection of 0.5 mg glyceryl trinitrate has been sufficient to counteract rebound arterial hypertension instantaneously.

A subsequent study by several members of the same group investigated the dose response to adenosine in 5 patients, 4 adults and 1 child, undergoing AVM embolization with adenosine induced asystole. In total 29 injections of adenosine were performed across 7 procedures. Between two and five injections of adenosine were performed to estimate the optimal dose required with the initial test bolus being between 0.25–0.35 mg/kg. Subsequent doses were escalated by 10–20 mg after an interval of 3–10 min until a 20–30 s stable MAP reduction to 25–30 mm Hg. To counteract the rebound post-adenosine hypertension a sodium nitroprusside infusion (≈1 µg/min/kg) was titrated to lower the baseline MAP by about 10%. The mean adenosine dose was 0.98 ± 0.4 mg/kg with a range between 0.24–1.76 mg/kg (6–90 mg) across the participants. The duration of asystole, defined as longest R‑R interval observed after the adenosine injection, was 8 ± 3 s. The duration of MAP <30 mm Hg and MAP <50 mm Hg was 18 ± 12 s and 50 ± 29 s, respectively. The authors noted a linear relationship between the dose and the duration of asystole, MAP <30 mm Hg and <50 mm Hg. In our experience we use a fixed dose of 36 mg of adenosine injected directly into a venous catheter the tip being placed in the right atrium, which induces asystole of 10–20 s in the majority of patients and as such we do not typically alter the dose or carry out test bolus injections. One advantage of AVM embolization with cardiac standstill is the global effect on flow within the AVM. The use of microballoons to decrease flow from a particular artery may have unpredictable consequences on the flow within the remainder of the AVM that receives a different arterial supply. This is not the case with cardiac standstill since the flow across all arterial inputs is reduced.

Rapid ventricular pacing (RVP) is a potential alternative to adenosine induced asystole and became popular in the context of transcatheter aortic valve replacement (TAVR) [16]. We have limited experience with RVP since the logistics in the setting of AVM embolization are more complex than for adenosine induced asystole. The main advantage of RVP is the possible longer duration (up to 30 s) of reduced cardiac output. The contraindications for adenosine (e.g., chronic obstructive pulmonary disease) do not apply. The circulatory effect, however, is less distinct. The RVP is typically producing a systolic blood pressure ≤50 mm Hg, but no standstill of the bloodstream. The glue injection during adenosine induced asystole on the contrary shows no flow-dependent propagation of the column of polymerizing embolic agent, which allows avoiding venous passage and occlusion.

Our study has several limitations including those inherent to a retrospective study. Although the number of patients in this series represents the largest case series to date using this method the overall numbers are still small. Similarly, several patients are awaiting delayed angiography to determine if a residual shunt is present.

## Conclusion

Adenosine induced cardiac standstill represents a viable treatment strategy in high flow AVMs or AV shunts that carries a low risk of mortality and permanent neurological deficit.
